# Supplementary material for: Empirical methods for controlling false positives and estimating confidence in ChIP-Seq peaks
Source: BMC Bioinformatics. 2008 Dec 5;9:523. doi: 10.1186/1471-2105-9-523 (PMC2628906; doi:10.1186/1471-2105-9-523)
Supplement: Additional File 1 — A variety of html documents from the USeq web site detailing the available applications, their best usage, output file type descriptions, command line menus, etc. [file 1471-2105-9-523-S1.zip › USeqUserGuides/JavaDoc.zip!/JavaDoc/allclasses-frame.html]

All Classes


**All Classes**
  

AccountManagement
  
ADFDataBaseSplitter
  
AggregatePlots
  
AlignedBaseCounter
  
AlignerInputForm
  
AlignerInputPanel
  
AlignerPreferences
  
AlignerThread
  
Alignment
  
Alignment
  
AlignPanel
  
AlignParams
  
Amplicon
  
AnalysisBean
  
AnalysisGroupBean
  
AnalysisProtocolBean
  
AnalysisTypeBean
  
AnnoSpecParams
  
AnnotateRegions
  
AnnotateRegionsWithGeneList
  
Annotation
  
AntibodyBase
  
AntibodyBean
  
AntibodyModify
  
AntibodyPrep
  
AntibodySpreadSheet
  
AntibodyViewPreferences
  
Archive
  
ArrayPrinter
  
AverageACels
  
AverageDoubles
  
AveragePCels
  
BackGroundOligo
  
Bar2Gr
  
BarParser
  
BaseCount
  
BaseRead
  
Bed2Gr
  
BestWindowScoreExtractor
  
Bin
  
Bin
  
BinaryBooleanReaderWriter
  
BindingPeak
  
BindingRegion
  
BindingRegionComparator
  
BindingRegionGraphPrinter
  
BindingRegionsToGff3
  
BindingRegionsToIntervals
  
BL2Seq
  
BL2SeqHit
  
Blast
  
Blast
  
BlastCompompositeGenerator
  
BlastFilterResult
  
BlastMatcher
  
BlastParser
  
BlastResult
  
Box
  
BrowseButtonAction
  
CalcBkgrndRatios
  
CalcGCAndTransform
  
CalcGCRatiosFromIntervals
  
CalculateBackgroundStats
  
CalculateGeneExtensions
  
Cap3Parser
  
CelFileConverter
  
CelFileQualityControl
  
CelFileStats
  
CelLine
  
CellType
  
CellTypeBean
  
CelMapper
  
CelMasker
  
CelPHistogram
  
CelProcessor
  
ChartFrame
  
ChromosomeIndex
  
ChromSet
  
Cluster
  
ClusterDrawFrame
  
ClusterDrawPanel
  
CollapseTPMap
  
CombineXYSeries
  
ComparatorEnrichedRegionPosition
  
ComparatorEnrichedRegionScore
  
ComparatorInfoPoint
  
ComparatorMultiSetQuantile
  
ComparatorPointPosition
  
ComparatorQuantile
  
ComparatorSmoothingWindowPosition
  
ComparatorSmoothingWindowScore
  
ComparatorSmoothingWindowTwoScore
  
CompressFasta
  
ConservedSeqs
  
Console
  
Contact
  
ContactBean
  
ControlGene
  
ControlStats
  
ConvertAgilentData
  
ConvertBars2TPMapCel
  
ConvertFasta2GCBoolean
  
ConvertFasta2RealSeqBoolean
  
ConvertGeoData
  
ConvertNimblegenNDF2TPMap
  
ConvertNimblegenPAIR2Cela
  
ConvertTPMapsToFeatures
  
ConvertTVSToCelps
  
Coordinate
  
CoordinateExtractor1lq
  
Correlate
  
CorrelateGrFiles
  
CorrelationMaps
  
CorrelationWindow
  
CpG
  
CronArchive
  
CubicSpline
  
DataLine
  
DBUtil
  
DefinedRegionScanSeqs
  
DeleteMeNow
  
Delme
  
Delme2
  
DelmeAligner
  
DelmeTest
  
DestypeIntensityCalculator
  
DividePointData
  
DmelCGNameCoordinatePrinter
  
DmelRel4Extractor
  
DrosGeneCollFileParser
  
ElandExportLine
  
ElandMultiParser
  
ElandParser
  
ElandSequenceParser
  
Email
  
EnrichedRegion
  
EnrichedRegionMaker
  
Enzyme
  
ExonIntron
  
ExonScoreExtractor
  
ExportExons
  
ExportIntergenicRegions
  
ExportIntervalData
  
ExportIntronicRegions
  
ExpressedGene
  
ExpressionScores2CoordinatesViaGFF
  
ExtractGffFeatures
  
ExtractNonOverlappingWindows
  
ExtractSaveSgrScores
  
ExtractScoresForRoc
  
ExtractWords
  
Fasta
  
FDRVsQValues
  
FDRWindowConverter
  
Feature
  
FetchGenomicSequences
  
FetchIndexedSequence
  
FetchSeqsQualsByName
  
FileCrossFilter
  
FileFocusListener
  
FileInputForm
  
FileInputPanel
  
FileJoiner
  
FileMatchJoiner
  
FileSize
  
FileSplitter
  
FileTrimmer
  
FilterAgilentProbeFile
  
FilterPointData
  
FilterTPMapByRegions
  
FindNeighboringGenes
  
FindSubBindingRegions
  
Flash
  
GadFlyGffExtractor
  
GATAFrame
  
GATAligner
  
GATAParams
  
GATAPlotter
  
GATAUtil
  
GCRatio
  
Gene
  
GeneBean
  
GeneCentricCNVParser
  
GeneGroup
  
GeneNameCounter
  
GeneRep
  
GeneRepGlyph
  
GenericFeature
  
GenericGlyph
  
GenomeBean
  
GenomeFASTASplitter
  
GenomicRegion
  
GenomicRegionGlyph
  
Gff2ToBindingRegions
  
Gff2ToIntervals
  
Gff3Feature
  
Gff3Parser
  
GffFeature
  
GffParser
  
GlyphPanel
  
Gr
  
Gr2Bar
  
Graph
  
GrComparator
  
GrGraph
  
Gump
  
GumpApplicationPanel
  
GumpDrawFrame
  
GumpResults
  
GumpResultsScrollPane
  
GumpToolPanel
  
HeatMapMaker
  
HeatMapMakerPosNeg
  
HierarchicalClustering
  
Histogram
  
HistogramSgrOrGrFile
  
HouseBean
  
HTML
  
IdNameMatch
  
IndexedSequence
  
IndexFastas
  
IndexRegions
  
InefficientPseudoMedian
  
Info
  
InfoPoint
  
IntensityFeature
  
IntensityPrinter
  
IntersectBindingPeaks
  
IntersectKeyWithRegions
  
IntersectLists
  
IntersectRegions
  
Interval
  
IntervalComparator
  
IntervalDrawFrame
  
IntervalDrawPanel
  
IntervalFilter
  
IntervalGFFPrinter
  
IntervalGraphPrinter
  
IntervalMaker
  
IntervalPlotter
  
IntervalReportPrinter
  
IO
  
Job
  
JohnsonToTag
  
JQSub
  
LabGroup
  
LabGroupBean
  
LabGroupUserType
  
LinearRegression
  
LoadChipSetIntervalOligoInfo
  
LoadedOligo
  
LoadIntervalOligoInfo
  
LocalAlignment
  
Login
  
Login
  
LoginBean
  
LoginBean
  
Logout
  
LowerCase2Xs
  
MakeAllWindows
  
MakeBioTools
  
MakeChromosomeSets
  
MakeSpliceJunctionFasta
  
MakeTranscriptome
  
MakeUSeq
  
MapControlIntensities
  
MapFeature
  
MapSplitter
  
Marker
  
MarkerBean
  
MaskExonsInFastaFiles
  
MD5
  
MD5Crypt
  
MemeMotif
  
MemeParser
  
MemeR
  
MemeResults
  
MergeColumns
  
MergeFastas
  
MergeRegions
  
MergeWindowArrays
  
MethodTester
  
MethylationParser
  
MiRNA
  
MiRNATarget
  
Misc
  
MotifHit
  
MotifScanner
  
MotifSearchResult
  
MultiAlignPanel
  
MultiFastaParser
  
MultiSetQuantile
  
MultiSGRPrinter
  
MultiWindowIntervalMaker
  
MummerMapper
  
NEBParser
  
NewAntibody
  
NewOligo
  
NewPlasmid
  
NewStrain
  
NormalizeAgilentData
  
NucleicAcid
  
Num
  
Oligo
  
OligoBase
  
OligoBean
  
OligoFlipper
  
OligoIntensityPrinter
  
OligoMatch
  
OligoModify
  
OligoSpreadSheet
  
OligoTiler
  
OligoUpload
  
OligoUploadBean
  
OligoViewPreferences
  
Option
  
Organism
  
OrganismBean
  
Organization
  
OverlapCounter
  
ParseAgilentData
  
ParseDecodeGenotypeData
  
ParseManipulateColumnDataForGeneSifter
  
ParseMiRNATable
  
ParseMLSHTML
  
ParsePatternedWindows
  
Parser1lq
  
ParserForGff
  
ParseSeqsFromUCSCGeneTable
  
ParseSgrsForParticularRegions
  
ParseSgrsForParticularScores
  
ParseWormBaseGffToUCSC
  
ParseWormMart
  
ParseXMLFile
  
Passwords
  
PatternMatch
  
PeakPicker
  
PeakPicker
  
PearsonCorrelation
  
PlasmidBase
  
PlasmidBean
  
PlasmidModify
  
PlasmidSpreadSheet
  
PlasmidUpload
  
PlasmidUploadBean
  
PlasmidViewPreferences
  
PlotFloatArrays
  
PlotterPreferences
  
Point
  
PointData
  
Positive
  
PositiveComparator
  
PosRegion
  
PossumToGFF
  
Preference
  
PrependColumn
  
Primer3Pick
  
Primer3Wrapper
  
PrintSelectColumns
  
ProcessedDataBean
  
ProcessedDataTypeBean
  
PWMScoreWindows
  
QCSeqs
  
QualityFileParser
  
Quantile
  
QuantileNormalization
  
RandomizeLabels
  
RandomRegions
  
RankedFloatArray
  
RankedSetAnalysis
  
RankedSetDrawFrame
  
RankedSetDrawPanel
  
RankSample
  
RankSampleIndexComparator
  
RankSampleValueComparator
  
RawDataBean
  
Read
  
ReadAlignParser
  
ReadZip
  
ReagentBean
  
REDigest
  
Reference
  
RefSeqAnnotationParser
  
Region
  
RegionComparator
  
Regions2Intervals
  
RenameRWSResults
  
RenameWords
  
RepeatMaskerToGFF
  
ReplaceWindowScores
  
RipInparanoidResults
  
RipMGIOrthologyResults
  
RocWindow
  
RocWindowScanner
  
RotateCelFile
  
S3CodeSamples
  
S3Manager
  
SamChromosomeData
  
Sample
  
SaveImageForm
  
SaveImagePanel
  
ScaleBar
  
ScanChip
  
ScanChromosomes
  
ScanChromosomesCNV
  
ScanGenes
  
ScanSeqs
  
ScatterDrawFrame
  
ScatterDrawPanel
  
ScatterPlot
  
ScoreChromosomes
  
ScoredCoordinate
  
ScoredGene
  
ScoredRegionsFormatFixer
  
ScoreIntervals
  
ScoreParsedBars
  
ScoreSequences
  
Search
  
SearchBean
  
SearchHit
  
Segment
  
SelexParams
  
SelexSeqParser
  
Seq
  
SeqFiles
  
SeqRead
  
Seqs
  
SetNumberIntervalMaker
  
Sgr
  
Sgr2Bar
  
SgrBacCounter
  
SgrComparator
  
ShiftPositionsInUCSCGeneLines
  
SimpleBlast
  
SimpleTextWindow
  
Simulator
  
Sliders
  
SmoothData
  
SmoothingWindow
  
SmoothingWindowInfo
  
Spammer
  
SpearmanCorrelation
  
SpeciesBean
  
SplineScalar
  
SplitWindowArraysByChromosome
  
SQL
  
StartStop
  
StartStop
  
Stat1lq
  
StatBarFiles
  
StatFlag
  
StatIntensityFeatureArray
  
StatMethylationData
  
StatTPMap
  
StatTPMapControlSeqs
  
StrainBase
  
StrainBean
  
StrainModify
  
StrainPreference
  
StrainSpreadSheet
  
StrainUpload
  
StrainUploadBean
  
StrainViewPreferences
  
StripZeros
  
SubSamplePointData
  
SubSeq
  
SubtractOneFromEachColumn
  
SubtractRegions
  
SubWindow
  
Swing
  
SynonymMatching
  
T2
  
T2Parameter
  
TableHeading
  
Tag2Point
  
TASSignalChromFileSplitter
  
TestInBioTools
  
TestScanChip
  
TextFrame
  
TextParser
  
TextPValOrSig2Gr
  
TextWindow
  
ToolsFrame
  
TPMapDensityReducer
  
TPMapDuplicateFilter
  
TPMapFeature
  
TPMapLine
  
TPMapOligoBlastFilter
  
TPMapProcessor
  
TPMapSort
  
TPMapStripHighLowGC
  
Transcript
  
TransformRatios
  
TransGroup
  
TransGrpGlyph
  
Translation
  
UCSCGeneLine
  
UCSCGeneLineComparator
  
UCSCGeneLineComparatorScore
  
UCSCGeneModelTableReader
  
UseDilution
  
UserBean
  
UserBean
  
UserRegistration
  
UserRegistrationBean
  
UserTypeBean
  
Util
  
Util
  
Util
  
UtilMeme
  
UtilSelex
  
VirtualCel
  
VirtualCelFrame
  
VirtualCelPanel
  
VisibilityBean
  
Wig2Bar
  
WilcoxonRankSumTest
  
WilcoxonSample
  
WilcoxonSamplePair
  
WilcoxonSignedRankTest
  
Window
  
WindowBlockMaker
  
WindowBlockMakerTwoColor
  
WindowComparator
  
WindowHistogram
  
WindowMaker
  
Windows2HeatMapSgr
  
WindowScoreSmoother
  
Xamer
  
XamerCounter
  
XML
  
ZipAndDelete
